# Effect of a Multiorgan Focused Clinical Ultrasonography on Length of Stay in Patients Admitted With a Cardiopulmonary Diagnosis: A Randomized Clinical Trial

**DOI:** 10.1001/jamanetworkopen.2021.38228

**Published:** 2021-12-21

**Authors:** Ximena Cid-Serra, Alistair Royse, David Canty, Douglas F. Johnson, Andrea B. Maier, Tim Fazio, Doa El-Ansary, Colin F. Royse

**Affiliations:** 1 Department of Surgery, The University of Melbourne, Melbourne, Victoria, Australia; 2 Department of Medicine and Community Care, The Royal Melbourne Hospital, Melbourne, Victoria, Australia; 3 Department of Surgery, The Royal Melbourne Hospital, Melbourne, Victoria, Australia; 4 Department of Medicine, Monash University, Melbourne, Victoria, Australia; 5 Department of Anaesthesia and Pain Management, The Royal Melbourne Hospital, Melbourne, Victoria, Australia; 6 Department of Anesthesia and Perioperative Medicine, Monash Health, Melbourne, Victoria, Australia; 7 Department of General Medicine, The University of Melbourne, Melbourne, Victoria, Australia; 8 Department of Human Movement Sciences, @AgeAmsterdam, Amsterdam Movement Sciences, Vrije Universitet, Amsterdam, The Netherlands; 9 Department of Medicine and Aged Care, The Royal Melbourne Hospital, Melbourne, Victoria, Australia; 10 Health Intelligence Unit, Melbourne Health, Melbourne, Victoria, Australia; 11 Department of Medicine and Radiology, Melbourne Medical School, University of Melbourne, Melbourne, Victoria, Australia; 12 Department of Nursing and Allied Health, Swinburne University of Technology, Hawthorn, Melbourne, Victoria, Australia; 13 Department of Surgery, School of Medicine, University of Melbourne, Melbourne, Victoria, Australia; 14 Clinical Research Institute, Westmead Private Hospital, Westmead, Sydney, New South Wales, Australia; 15 Pain Management, The Royal Melbourne Hospital, Melbourne, Victoria, Australia; 16 Outcomes Research Consortium, The Cleveland Clinic, Cleveland, Ohio

## Abstract

**Importance:**

There are accumulating data about the utility of diagnostic multiorgan focused clinical ultrasonography (FCU) in the assessment of patients admitted with cardiopulmonary symptoms.

**Objective:**

To determine whether adding multiorgan FCU to the initial clinical evaluation of patients admitted with cardiopulmonary symptoms reduces hospital length of stay, hospital readmissions, and in-hospital costs.

**Design, Setting, and Participants:**

This is a prospective, parallel-group, superiority, randomized clinical trial with a 1:1 allocation ratio. The study was conducted at The Royal Melbourne Hospital, a tertiary public hospital located in Melbourne, Victoria, Australia. Adults aged 18 years or older admitted to the internal medicine ward with a cardiopulmonary diagnosis were enrolled between September 2018 and December 2019 and were followed up until hospital discharge. Data analysis was performed from August 2020 to January 2021.

**Interventions:**

The intervention involved an internal medicine physician–performed heart, lung, and 2-point vein compression FCU in addition to standard clinical evaluation.

**Main Outcomes and Measures:**

The primary outcome was the difference in the mean length of hospital stay, defined as the number of hours from admission to the internal medicine ward to hospital discharge. A difference of 24 hours was defined as clinically important. Secondary outcomes included hospital readmissions at 30 days and hospital care costs.

**Results:**

A total of 250 participants were enrolled and 2 were excluded, leaving 248 participants (mean [SD] age, 80.1 [11.0] years; 121 women [48.7%]) in the final analysis. There were 124 patients in the intervention group and 124 patients in the control group. The most common initial diagnoses were acute decompensated heart failure (113 patients [45.5%]), pneumonia (45 patients [18.1%]), and exacerbated chronic pulmonary disease (32 patients [12.9%]). The length of hospital stay was 113.4 hours (95% CI, 91.7-135.1 hours) in the FCU group and 125.3 hours (95% CI, 101.7-148.8 hours) in the control group (*P* = .53). The 30-day readmission rate was not different between groups (FCU vs control, 20 of 124 patients [16.1%] vs 15 of 124 patients [12.0%]), nor were total in-hospital costs (FCU vs control, A$7831.1 [95% CI, A$5586.1-A$10 076.1] vs A$7895.7 [95% CI, A$6385.9-A$9.405.5]).

**Conclusions and Relevance:**

In this randomized clinical trial, adult patients admitted to an internal medicine ward with a cardiopulmonary diagnosis, who underwent multiorgan FCU of the heart, lungs, and lower extremities veins during their initial clinical assessment, did not have a shorter hospital length of stay by more than 24 hours, compared with patients who received standard care.

**Trial Registration:**

Australian New Zealand Clinical Trials Registry Identifier: ACTRN12618001442291

## Key Points

**Question:**

Does integrating multiorgan focused clinical ultrasonography with clinical evaluation of patients admitted with cardiopulmonary symptoms reduce the hospital length of stay?

**Findings:**

In a randomized clinical trial of 250 participants allocated to undergo multiorgan focused clinical ultrasonography or standard management at admission to the hospital, the hospital length of stay was not different compared with control patients.

**Meaning:**

These findings suggest that integration of multiorgan focused clinical ultrasonography with the initial evaluation does not reduce hospital length of stay by more than 24 hours.

## Teaser

This randomized clinical trial examines whether adding multiorgan focused clinical ultrasonography (FCU) to the initial clinical evaluation of patients admitted with cardiopulmonary symptoms reduces hospital length of stay, hospital readmissions, and in-hospital costs.

## Introduction

Internal medicine physicians traditionally initiate their clinical evaluation on the basis of the patient’s medical history and physical examination findings. However, clinical evaluation alone is frequently inaccurate in determining the correct diagnosis,^1,2,3,4^ requiring further investigations, such as chest radiograph, blood tests, and, in some cases, echocardiography or computed tomography. Incorporating a bedside ultrasonography performed by the same treating physician increases the precision of the initial clinical evaluation.^5^

Focused clinical ultrasonography (FCU) has been shown to be a reliable and accurate test compared with the imaging reference standard.^6,7,8,9,10,11,12,13,14,15,16^ FCU has the additional advantage of being free of ionizing radiation and performed in real-time at the patient’s bedside, increasing the speed and accuracy of the initial diagnosis^17^ and avoiding the cost, time, and potential risk of transferring patients to other locations for investigations.

There are accumulating data about the utility of FCU in the assessment of patients with cardiorespiratory symptoms. Lung ultrasonography alone or combined with focused cardiac ultrasonography is highly accurate^18,19,20,21,22,23,24,25,26,27,28,29^ and superior to a chest radiograph^30^ in distinguishing acute decompensated heart failure (ADHF) from other causes of dyspnea. Lung ultrasonography can quickly identify alternative diagnoses such as pneumonia and pleural effusion with sensitivity and specificity greater than 93% for both lung diseases.^12,14^ In patients with chronic obstructive pulmonary disease (COPD) presenting with dyspnea, normal lung ultrasonography findings support a diagnosis of COPD exacerbation with 89% sensitivity and 97% specificity.^21,31^ Multiorgan FCU involving the heart, lungs, and lower extremity veins has been reported to have 90% sensitivity and 86% specificity for diagnosing pulmonary embolism (PE).^32^ This multiorgan FCU has demonstrated superiority to standard diagnostics tests alone for establishing the correct diagnosis within 4 hours in patients with respiratory symptoms presenting to the emergency department.^17^

The rationale behind the use of FCU is to improve the diagnostic fidelity and speed of clinical evaluation. An additional premise is that prompt and appropriate management will be initiated and guided by the FCU findings, which, in turn, could improve the workflow, reduce the time for definitive diagnosis, potentially rationalize imaging and other investigations, and could lead to reduced hospital length of stay (LOS) and cost. However, there is limited information on how FCU affects the clinical decision-making process in the internal medicine setting.^33,34,35,36^ Moreover, the information available on the impact of FCU on the hospital LOS of internal medicine inpatients derives from 2 vastly different randomized studies. Lucas et al^36^ studied internal medicine inpatients referred for standard echocardiography and did not find a difference between groups by adding focused cardiac ultrasonography. Conversely, Mozzini et al,^37^ using repeated lung ultrasonography in patients with ADHF admitted to the internal medicine ward, reported a significant reduction of the hospital LOS of 1 day.

The primary aim of our study was to determine whether a heart, lung, and lower extremity vein FCU performed within 24 hours of admission reduces the hospital LOS by at least 24 hours in patients admitted to internal medicine wards with a cardiopulmonary diagnosis. The secondary aims were to evaluate the impact of this multiorgan FCU on the 30-day hospital readmission rates and in-hospital costs.

## Methods

This study was a prospective, randomized, parallel-group, superiority trial performed at Royal Melbourne Hospital in Victoria, Australia. Participants were recruited between September 2018 and December 2019 and were followed up until hospital discharge.

Before participant enrollment, the study was approved by The Melbourne Health Research Ethics Committee and was conducted following the Declaration of Helsinki.^38^ The protocol was published during the initial recruitment phase (Supplement 1) with no changes to methods after the trial commencement. This study followed the Consolidated Standards of Reporting Trials (CONSORT) reporting guideline.

Eligible participants were all adults aged 18 years or older admitted to the internal medicine ward within 24 hours, with a preliminary cardiopulmonary diagnosis determined by the treating internal medicine physician. Examples of cardiopulmonary conditions included ADHF, acute coronary syndrome, PE, pneumonia, exacerbated COPD, asthmatic crisis, cardiogenic syncope, interstitial pulmonary disease, cardiac valve disease, pleural effusion, pericardial effusion, or undifferentiated dyspnea. Exclusion criteria were standard echocardiography performed in the 4 weeks before hospital admission, computed tomography chest scan performed during the current hospital admission, a requirement for infectious isolation, or the inability to obtain consent from the patient or their responsible person.

After written informed consent was obtained from the participant or next of kin, participants were enrolled and randomly allocated to the intervention or control group (1:1). The allocation sequence was generated by computer software. Sealed and double-layered envelopes were used for concealment. The allocation was not blinded to the patient or treating health care team but to the person adjudicating the primary outcome.

During the first 24 hours from the time of admission, participants in the intervention group underwent FCU of the heart, lung, and lower extremity veins performed at the patient’s bedside by an internal medicine physician (X.C.-S.) with the certification of iHeartScan^39,40,41^ and iLungScan^42^ courses from the University of Melbourne. All FCU images and reports were reviewed by a senior expert in FCU (C.F.R., D.C., or A.R.) before providing the FCU information to the treating team. A standardized report summarizing the main FCU findings was given to the treating team without giving any advice on management (eFigure in Supplement 2).

Participants in the control group received standard care, which did not include multiorgan FCU. In this group, clinical decisions were based on clinical evaluation and other further investigations. Control group participants were not excluded from undergoing ultrasonography examinations performed by cardiology or radiology departments. In both groups, the treating team completed a clinical assessment form describing the patient’s hemodynamic state, physical examination findings, the most likely diagnosis, further investigations, medical referrals, and the type of treatment prescribed to treat the cardiopulmonary condition.

Ultrasonography equipment used included an X-Porte ultrasonography machine (Sonosite; Bothwell), using a 1- to 5-MHz phased-array transducer for heart and lungs and a 6- to 13-MHz linear ultrasonography transducer for deep veins of the lower limb. The scanning followed the iHeartScan^39,40,41^ and iLungScan^42^ protocols, which were designed and validated by the Ultrasonography Education Group of the University of Melbourne, and the 2-point technique for assessing the lower extremity veins.^43,44^ The definition of substantial cardiac abnormalities in this protocol correlates with moderate-to-severe abnormalities in a standard echocardiography. The following cardiac variables were assessed on the basis of 2-dimensional images and color flow Doppler ultrasonography: volume and systolic function of the left and right ventricles, left atrial filling pressure based on the interatrium septum movement,^45^ regurgitation or stenosis of the cardiac valves, presence or absence of pericardial effusion, diameter and collapsibility index of inferior vena cava, and hemodynamic state assessment.^39^ The following lung abnormalities were assessed: alveolar or interstitial syndrome, lung collapse, lung consolidation, pneumothorax, and pleural effusion, including estimation of its volume.^42^ Proximal leg veins were scanned using the 2-point technique evaluating the femoral and popliteal veins’ collapsibility at the level of the groin and popliteal fossa.^43^

### Outcomes

The primary outcome was the difference in the mean hospital LOS between groups, defined as the number of hours from hospital admission to discharge. The data were obtained from the hospital Health Intelligence Unit by a third person (T.F.) blinded to group allocation. A clinically meaningful effect was defined as a 24-hour shorter duration. Secondary outcomes were the proportion of patients unexpectedly readmitted during the next 30 days after hospital discharge and health care costs determined by the hospital Health Intelligence Unit (blinded to group allocation) in multiple categories, including staff cost, bed days, and investigations.

### Sample Size

A sample size of 122 participants in each group was calculated according to a 2-sided *t* test of the log-transformed LOS (before the study, the mean LOS of the patients admitted to the internal medicine ward at the Royal Melbourne Hospital was 103 hours), a clinically meaningful effect on LOS defined as greater than or equal to 24 hours, and using a power of 80% and α = .05. This number was rounded up to a total of 250 participants to compensate for potential withdrawals.

### Statistical Analysis

The primary outcome, hospital LOS in hours, was analyzed following an intention-to-treat approach and using the 2-sided *t* test on log-transformed data to normalize skewed data. A cutoff of 30 days was applied to avoid the effect of excessively extended hospital stays. Significance was defined as *P* < .05. Death in the hospital was expected to be rare; therefore, death was treated as hospital discharge for the primary analysis for patients who died in the hospital. Patients with missing data were not included in the primary statistical analysis.

Secondary outcomes were analyzed using parametric or nonparametric tests according to the type of data. All estimates were reported with 95% CIs. Health costs data were log-transformed and analyzed using the 2-sided *t* test. Thirty-day readmission data were analyzed using a 2-sided Fisher exact test. An exploratory analysis was performed to compare the first half vs the second half of the participants on the basis of our hypothesis that there would be a lead-in period where the FCU findings may not be incorporated by the treating team, whereas as the physicians became more familiar with FCU, the findings would be more trusted and more likely to be acted upon.

We used SPSS statistical software version 27 (IBM) for our analyses. Data analysis was performed from August 2020 to January 2021.

## Results

**Figure 1.**
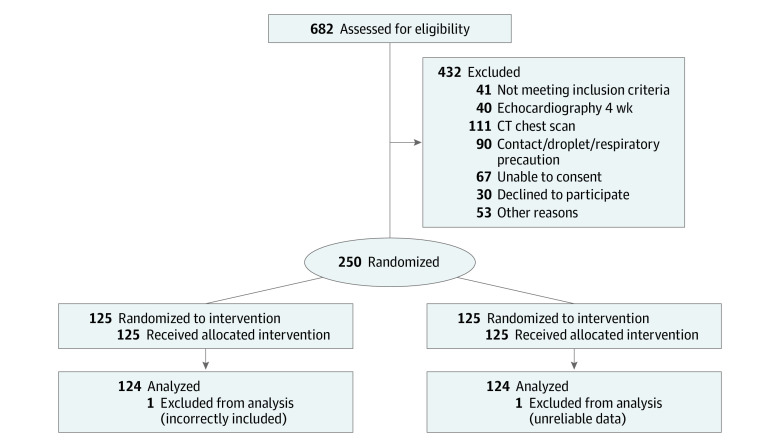
Study Enrollment Flowchart CT indicates computed tomography.

Of 682 patients screened for eligibility, 402 were excluded, 30 declined to participate, and 250 were enrolled. Reasons for exclusion are detailed in Figure 1. There was no crossover between groups; all the participants received the correct allocation. Two participants were excluded from the final analysis. One patient was excluded because their admission lasted longer than 24 hours at the time of recruitment, and 1 participant had unreliable data about the LOS because their identification number matched that of a different patient.

Demographic and clinical characteristics were similarly distributed among groups (Table 1). Of the 248 remaining participants (124 each in the intervention and control groups), 121 (48.7%) were women, and the mean (SD) age was 80.1 (11.0) years. Shortness of breath was the most common presenting complaint for 207 participants (83.4%). The most common initial diagnoses were ADHF (113 patients [45.5%]), lower respiratory tract infection or pneumonia (45 patients [18.1%]), and exacerbation of COPD (32 patients [12.9%]).

**Table 1.** Demographic and Baseline Clinical Data

Characteristic	Patients, No. (%) (N = 248)	
	FCU (n = 124)	Control (n = 124)
Age, mean (SD), y	80.1 (11.0)	79 (12.2)
Sex		
Female	67 (54.0)	54 (43.5)
Male	57 (46.0)	70 (56.5)
Body mass index, mean (SD)^a^	29.1 (8.1)	29.3 (7.0)
Vital signs at recruitment		
Systolic blood pressure, mean (SD), mm Hg	126.3 (16.7)	128.6 (18.8)
Heart rate, mean (SD), beats/min	77.7 (15.3)	78.4 (16.3)
Respiratory rate, mean (SD), breaths/min	20.1 (2.8)	20.3 (7.2)
O_2_ saturation, mean (SD), %	94.4 (8.0)	94.5 (7.5)
Oxygen therapy	35 (28.2)	25 (20.2)
Previous medical condition		
Hypertension	82 (66.1)	83 (66.9)
Chronic cardiac failure	65 (52.4)	60 (48.4)
Myocardial infarction	29 (23.4)	23 (18.5)
Cardiac valve disease	18 (14.5)	16 (12.9)
Arrhythmia	48 (38.7)	50 (40.3)
Chronic obstructive pulmonary disease	26 (21.0)	35 (28.2)
Asthma	9 (7.3)	15 (12.1)
Smoker	20 (16.1)	21 (16.9)
Diabetes	40 (32.3)	46 (37.1)
Chronic kidney disease	18 (14.5)	20 (16.1)
Stroke	8 (6.5)	15 (12.1)
Previous venous thromboembolism	16 (12.9)	14 (11.3)
Cancer	23 (18.5)	20 (16.1)
Hypothyroidism	17 (13.7)	17 (13.7)
Dementia	23 (18.7)	21 (16.9)
Charlson Comorbidity Index score, mean (SD)	5.47 (2.19)	5.33 (1.73)
Long-term medications		
Antihypertensive	80 (64.5)	72 (58.1)
Antiplatelet	40 (32.3)	44 (35.5)
Anticoagulation	40 (32.3)	40 (32.3)
β-blocker	46 (37.1)	48 (38.7)
Diuretics	57 (46.0)	59 (47.6)
Clinical presentation		
Shortness of breath	104 (83.9)	103 (83.1)
Lower limb edema	41 (33.1)	29 (23.4)
Chest pain	19 (15.4)	26 (21.0)
Palpitation	6 (4.8)	7 (5.6)
Cough	37 (29.8)	41 (33.1)
Fever	10 (8.1)	15 (12.1)
Altered level of consciousness	10 (8.1)	7 (5.7)
Most likely diagnosis at admission		
Acute decompensated heart failure	58 (46.8)	55 (44.4)
Lower respiratory tract infection or pneumonia	18 (14.5)	27 (21.8)
Exacerbated chronic obstructive pulmonary disease or asthmatic crisis	17 (13.7)	15 (12.1)
Pulmonary embolism	4 (3.2)	3 (2.4)
Acute coronary syndrome	3 (2.4)	6 (4.8)
Cardiogenic syncope	3(2.4)	4 (3.2)
Arrhythmia	5 (4.0)	1 (0.8)
Other	16 (12.9)	13 (10.5)

Abbreviation: FCU, focused clinical ultrasonography.

^a^ Body mass index is calculated as weight in kilograms divided by height in meters squared.

All 4 participants who died during the initial hospital admission (1.6%) had advanced chronic medical conditions. Multiorgan FCU findings in the intervention group are described in Table 2.

**Table 2.** Abnormal Ultrasonography Findings

Finding	Patients, No. (%) (n = 124)
Left ventricle systolic dysfunction	55 (44.4)
Aortic stenosis	30 (24.2)
Aortic regurgitation	14 (11.3)
Mitral stenosis	5 (4.0)
Mitral regurgitation	19 (15.3)
Tricuspid regurgitation	35 (28.2)
Pericardial effusion	6 (4.8)
Findings suggesting pulmonary embolism^a^	4 (3.2)
Bilateral interstitial or alveolar syndrome	21 (16.9)
Localized interstitial or alveolar syndrome	8 (6.5)
Lung consolidation	12 (9.7)
Pleural effusion	49 (39.5)
Lung collapse (atelectasis)	39 (31.5)
Deep venous thrombosis	5 (4.0)

^a^ Findings suggesting pulmonary embolism were right ventricle strain and deep venous thrombosis.

### Primary Outcome

**Figure 2.**
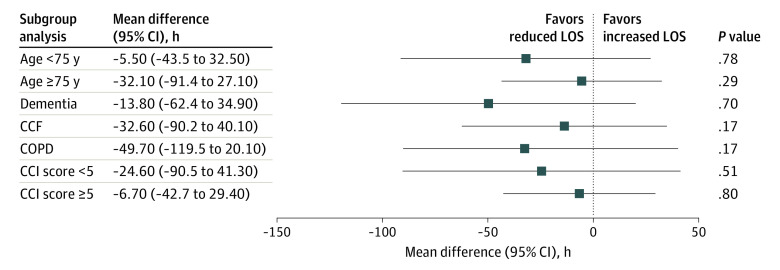
Subgroup Analysis of the Hospital Length of Stay (LOS) Graph shows difference in the mean hospital LOS between patients who underwent focused clinical ultrasonography and controls. A negative value (to the left) implies that focused clinical ultrasonography reduced the hospital LOS. CCF indicates chronic cardiac failure; CCI, Charlson Comorbidity Index; COPD, chronic obstructive pulmonary disease.

The mean hospital LOS was not different between groups (FCU vs control, 113.4 hours [95% CI, 91.7-135.1 hours] vs 125.3 hours [95% CI, 101.7-148.8 hours]; difference, 11.9 hours; *P* = .53). The exploratory analysis assessing the first and second half of the cohort separately showed a group separation exceeding 24 hours in the hospital LOS in the second half of recruitment. However, this was not significant. For the first half of the cohort, the LOS was 117 hours (95% CI, 85.5-148.5 hours) for the FCU group vs 116.4 hours (95% CI, 87.9-144.9 hours) for the control group (*P* = .89). In the second half of the cohort, the LOS was 109.8 hours (95% CI, 79.2-140.5 hours) for the FCU group vs 134.1 hours (95% CI, 95.9-172.4 hours) for the control group (*P* = .37). A further exploratory analysis was performed assessing the LOS of the FCU group vs controls in specific subgroups of the cohort (Figure 2).

### Secondary Outcomes and Harms

The incidence of hospital readmission within 30 days of discharge was not different between groups (20 of 124 patients in the FCU group [16.1%] vs 15 of 124 patients in the control group [12.0%]). There was no difference in the total hospital care costs between groups (FCU vs control, A$7831.1 [95% CI, A$5586.1-A$10 076.1] vs A$7895.7 [95% CI, A$6385.9-A$9405.5]; *P* = .79) (as of November 19, 2021, A$1 = US $0.73). Additional health cost data reported by category (bed stay, imaging test, pathology test, and others) is shown in Table 3. An exploratory subgroup analysis is reported in eTable 1 in Supplement 2.

**Table 3.** Hospitalization Costs for Each Group

Economic category	Costs, mean (95% CI), A$^a^		FCU vs control	
	FCU	Control	Mean absolute difference, A$	*P* value^b^
Internal medicine unit^c^	1281.9 (1021.8-1542.0)	1426.8 (1176.9-1676.7)	−144.9	.37
Internal medicine care^d^	4120.4 (3034.9-5205.9)	4264.5 (3471.8-5057.2)	−144.1	.56
Pathology tests	434.9 (325.6-544.2)	447.6 (326.2-569.0)	−12.7	.52
Imaging tests	196.0 (129.1-262.9)	163.9 (111.4-216.4)	32.1	.65
Other^e^	1808.2 (997.0-2619.5)	1616 (1199.0-2032.9)	192.3	.96
Total	7831.1 (5586.1-10 076.1)	7895.7 (6385.9-9405.5)	−64.6	.79

Abbreviation: FCU, focused clinical ultrasonography.

^a^ As of November 19, 2021, A$1 = US $0.73.

^b^ *P* value was calculated by comparing the means of the logarithmically transformed data in each group.

^c^ Refers to the unit’s cost associated with the physical stay.

^d^ Refers to the cost of being the patient’s primary treating team (physician salary) regardless of the unit where the patient was admitted or transferred during the hospital stay.

^e^ Includes various costs, such as emergency department, intensive care, allied health system, pharmacy, theater, meals, interpreter, and others.

We monitored the appearance of skin bruising and patient falls during the procedure or during patient transference from the chair to the bed to perform the examination. None of these events occurred.

We compared FCU reports against the reports of reference standard imaging tests when available. Of 124 participants in the FCU group, 18 had standard echocardiography during that hospitalization or in the following 3 months. The accuracy of FCU compared with the standard echocardiography is described in the eTable 2 in Supplement 2. FCU showed the lowest precision for right ventricle failure (75% sensitivity and 79% specificity) and the highest accuracy for significant aortic stenosis and pericardial effusion (100% sensitivity and 100% specificity for both cardiac abnormalities). A computed tomography pulmonary angiogram or a ventilation-perfusion scan confirmed the diagnosis in 4 patients for whom FCU findings suggested PE.

## Discussion

In this randomized clinical trial, a multiorgan FCU examination integrated with clinical evaluation within 24 hours of admission in patients with a cardiopulmonary diagnosis in an internal medicine unit did not reduce the hospital LOS by at least 24 hours. The mechanism for reducing hospital LOS by performing a multiorgan FCU is that earlier correct diagnosis will lead to better-informed management decisions, appropriate further investigations, and correct initial treatment. Furthermore, resolution of symptoms may lead to faster deescalation of therapy and earlier discharge.

FCU is ideally performed by the treating physician during the initial presentation so that decisions can be made in real time. Our study deviated from the ideal in that a trained physician conducted all the examinations and reported findings to the treating team. We anticipated that the impact of FCU on the hospital LOS would increase in the second half of the trial with the improvement of the clinicians’ ability to incorporate the FCU findings into their clinical evaluation and act accordingly. However, we did not find a significant difference in the LOS between groups in the second half of the cohort.

We performed an exploratory analysis assessing the effect of this multiorgan FCU on the hospital LOS of specific subgroups. We were particularly interested in participants with dementia, who frequently cannot provide a reliable history, and in individuals with COPD who present with cardiopulmonary symptoms and for whom the differential diagnoses are usually broad (exacerbated COPD, ADHF, PE, or pneumonia). However, in the subgroups analyzed, the differences in the LOS between FCU and controls were not significant.

In contrast to our study, Mozzini et al^37^ found a reduction of 1 day of the hospital LOS with lung ultrasonography compared with controls among patients admitted to internal medicine units with ADHF. The intervention used by Mozzini et al^37^ was a lung ultrasonography performed at multiple time points with the primary purpose of guiding diuretic therapy, which differs from our initial and onetime diagnostic ultrasonography examination. Previous randomized clinical trials reporting the effect on the hospital LOS of an initial FCU performed in the emergency department have shown opposite results. In trauma patients, the addition of FCU reduced the hospital LOS by 27%.^46^ However, in nontrauma patients presenting with undifferentiated shock, FCU did not affect the hospital LOS.^47^ Similarly, Laursen et al^17^ did not find a difference in the hospital LOS between groups with the addition of FCU performed in the emergency department for patients presenting with respiratory failure.

The hospital LOS predominantly influences in-hospital health costs. Therefore, it is not surprising that we did not find a substantial difference in the total in-hospital health costs between groups. This study was conducted in a department where the implementation of FCU was in a very early phase. During this initial stage, physician confidence with the FCU findings was variable, leading to further confirmatory investigations or keeping patients in the hospital for a longer time to assess incidental findings. This effect could potentially have led to higher costs, which did not occur.

In our study, FCU was not billed. The design did not look at patient benefits other than cost or hospital LOS. Further clinical outcomes should be considered in future studies, such as patient’s quality of life, patient’s recovery assessment, and any impact of misdiagnosis. The benefits of FCU should be weighed against the cost of implementation, such as the cost of equipment and training. Testa et al^48^ reported a cost-benefit analysis before and after the implementation of FCU in an internal medicine department. They described a breakpoint at 734 ultrasonography examinations estimated to be achieved in 406 days, after which the revenues exceed the incurred costs.^48^

Our cohort did not report any harm related to the use of multiorgan FCU. Readmissions in the following 30 days were evaluated as a safety parameter. Interventions that reduce the LOS at the hospital may increase the rate of readmissions due to premature discharges, which did not occur.

### Limitations

This study has several limitations. First, the cohort was mainly composed of older people for whom the acute medical condition may not be the only determinant of the hospital LOS. Multiple other variables can delay discharge, such as social problems or bed unavailability in rehabilitation centers, leading to wide 95% CIs in the LOS. We defined a priori that a clinically meaningful difference in LOS was 24 hours. This definition was arbitrary, and a shorter LOS difference, such as 12 hours, may be meaningful.

A single operator performed all multiorgan FCU examinations. The study deviated from an ideal practice whereby the treating physician would perform the FCU and integrate the findings into their clinical evaluation in real time. Furthermore, FCU is a dynamic assessment and should ideally be repeated after management changes or if the patient’s condition deteriorates.

In addition, although the protocol was designed to improve internal validity, our study was conducted in a single institution, which weakens external validity. When designing future studies, we recommend a pragmatic multicenter trial where the treating physicians perform the FCU. For institutions where FCU is not usual, we recommend that inclusion into any trial occurs after sufficient training and experience.

Exploratory analyses were conducted to identify potential effect size and to help power future studies. The reduced samples size for subgroups reduced the statistical power for comparisons, and a lack of significant difference in the presence of apparent large effect size is likely to represent a type II error.

## Conclusions

Adding multiorgan FCU to the initial clinical assessment compared with standard care did not reduce the hospital LOS among patients admitted with cardiopulmonary diagnoses to this internal medicine unit. Although there was a difference of 11.9 hours in the mean hospital LOS between groups, the result was not significant according to the prespecified clinically meaningful difference.

## Notes

Supplement 1.
Trial Protocol

Supplement 2. **eFigure.** iLungScan^TM^ and iHeartScan^TM^ Report Filled With an Example **eTable 1.** Subgroup Analysis of the Effect of POCUS in the Health Costs **eTable 2.** Accuracy of Cardiac Clinical Ultrasound in 18 Patients Compared to Standard Echocardiography Performed by Cardiologists

Supplement 3.
Data Sharing Statement
