# Errors in Figure 2

**DOI:** 10.1001/jamanetworkopen.2022.11248

**Published:** 2022-04-15

In the Original Investigation titled “Effect of a Multiorgan Focused Clinical Ultrasonography on Length of Stay in Patients Admitted With a Cardiopulmonary Diagnosis: A Randomized Clinical Trial,”^1^ published December 21, 2021, there were errors in Figure 2, with the labels on the left-hand side appearing in the incorrect order. This article has been corrected.^1^
