# Supplementary material for: Effect of a Multiorgan Focused Clinical Ultrasonography on Length of Stay in Patients Admitted With a Cardiopulmonary Diagnosis: A Randomized Clinical Trial
Source: JAMA Netw Open. 2021 Dec 21;4(12):e2138228. doi: 10.1001/jamanetworkopen.2021.38228 (PMC8693211; doi:10.1001/jamanetworkopen.2021.38228)
Supplement: Supplement 1. — Trial Protocol [file jamanetwopen-e2138228-s001.pdf]

PROTOCOL

A randomized trial of focused cardiac, lung, and femoral and popliteal vein ultrasound on the length of stay in internal medicine admissions with a cardiopulmonary diagnosis – the IMFCU 1 study

Version: 1
Date: 20/05/2018

Author/s:
Prof Colin Royse
Dr Ximena Cid
Prof Andrea Maier
Prof Alistair Royse
Dr David Canty
Dr Douglas Johnson
Ms Lynda Tivendale

Sponsor/s:
Nil

CONFIDENTIAL

This document is confidential and the property of Melbourne Health. No part of it may be transmitted, reproduced, published, or used without prior written authorisation from the institution.

Statement of Compliance

This study will be conducted in compliance with all stipulation of this protocol, the conditions of the ethics committee approval, the NHMRC National Statement on ethical Conduct in Human Research (2007) and the Note for Guidance on Good Clinical Practice (CPMP/ICH-135/95).

TABLE OF CONTENTS

CONTENTS

Table of Contents 3

1. **Glossary of Abbreviations & Terms** 7

2. **Study Sites** 7

2.1 Study Location/s 7

3. **Introduction/Background Information** 7

3.1 Lay Summary 7

3.2 Introduction 8

3.3 Background information 8

4. **Study Objectives** 10

4.1 Hypothesis 10

4.2 Study Aims 10

4.3 Outcome Measures 10

5. **Study Design** 10

5.1 Study Type & Design & Schedule 10

5.2 Standard Care and Additional to Standard Care Procedures 13

5.3 Randomisation 13

5.4 Study methodology 13

6. **Study Population** 14

6.1 Recruitment Procedure 14

6.2 Exclusion Criteria 14

6.3 Consent 14

7. **Participant Safety and Withdrawal** 14

7.1 Risk Management and Safety 14

7.2 Handling of Withdrawals 14

7.3 Replacements 15

8. Statistical Methods 15

8.1 Sample Size Estimation & Justification 15

8.2 Power Calculations 15

8.3 Statistical Methods To Be Undertaken 15

9. Storage of Blood and Tissue Samples 15

9.1 Details of where samples will be stored, and the type of consent for future use of samples 15

10. Data Security & Handling 16

10.1 Details of where records will be kept & How long will they be stored 16

10.2 Confidentiality and Security 16

10.3 Ancillary data 16

11. Appendix 17

12. References 17

STUDY SYNOPSIS

(please provide brief information)

Title:	A randomised trial of focused cardiac, lung, and femoral and popliteal vein ultrasound on the length of stay in internal medicine admissions with a cardiopulmonary diagnosis – the IMFCU 1 study
Short Title:	IMFCU 1 study
Design:	RCT
Study Centres:	Melbourne Health - RMH campus
Hospital:	RMH
Study Question:	Does physician performed focused cardiac, lung, and femoral and popliteal vein ultrasound reduce length of stay by at least 24 hours, for internal medicine admissions with a cardiopulmonary diagnosis?
Study Objectives:	To identify whether focused cardiac, lung, and femoral and popliteal vein ultrasound performed within 24 hours of hospital admission reduced length of stay in a cohort of patients with a cardiopulmonary admission diagnosis.
Primary Objectives:	Length of stay ≥ 24 hours defined clinical significance
Secondary Objectives	Time and cost analysis on major investigations and diagnoses prior to establishment of the definitive diagnosis<br><br>Health economic analysis
Inclusion Criteria:	Participants will be considered for inclusion if they meet all of the following criteria:<br>• Adult participants (≥18 years) with a cardiopulmonary diagnosis.<br>• Admitted to a general medical unit at the Royal Melbourne Hospital<br>• Able to provide informed consent<br>• Expected to remain in hospital >24 hours.
Exclusion Criteria:	Participants will be excluded if they meet any of the following criteria:<br>• admitted >24 hours<br>• admitted for social rather than medical reasons

	• unable to provide consent<br>• contact or respiratory isolation<br>• transthoracic echocardiogram within 4 weeks prior to admission<br>• CT chest scan during the admission process prior to enrolment
Number of Planned Subjects:	250
Investigational product:	N/A
Safety considerations:	The focused cardiac, lung and vein ultrasound is non-invasive and does not present risk to the patient
Statistical Methods:	The outcome of interest, hospital length of stay (LOS), is expected to be skewed and hence will be log transformed for the purposes of analysis. Control data were available for Q4 2017, with a mean log based 10 LOS of 2.01 (equivalent to 103 hours on the original scale) and a standard deviation of 0.36. In this study censoring will not be applied to the LOS. The primary outcome will be tested using Student t-test on the log-transformed LOS data.<br><br>In order to detect a clinically important reduction in LOS of 24 hours for the ultrasound group, based on the expected control value above and a common standard deviation on the log scale, power of 0.8 and size 0.05, 122 participants are required in each of the two treatment groups, which will be rounded up to a total of 250 participants.
Subgroups:	Standard care: no focused ultrasound is performed. Diagnosis and management decisions will be recorded prospectively.<br><br>Intervention group: Within 24 hours of admission to hospital, the researcher will perform a focused cardiac, lung, and femoral and popliteal vein ultrasound. The findings will be revealed to the medical team after they have completed a diagnosis and management form. Thereafter, all management is directed by the treating medical team and specifically, the intervention will not provide any management guidelines.

1. GLOSSARY OF ABBREVIATIONS & TERMS

Abbreviation	Description (using lay language)
FCU	Focused cardiac ultrasound – a goal focused heart ultrasound (echocardiography) performed at the patient bedside
Hospital Admission	Time from acceptance of admission to hospital (if admitted via ED), or arrival in the ward (if directly admitted from the community)
LOS	Length of stay- the number of hours a patient remains in hospital

2. STUDY SITES

2.1 STUDY LOCATION/S

Site	Address	Contact Person	Phone	Email
The Royal Melbourne Hospital	Grattan St, Parkville	Prof Colin Royse	0408467548	Colin.royse@heartweb.com

3. INTRODUCTION/BACKGROUND INFORMATION

3.1 LAY SUMMARY

When patients are admitted to hospital, they undergo clinical evaluation by the doctor, and then may undergo tests. Clinical evaluation alone is known to be inaccurate in up to 50% of patients with suspected heart or lung disease. Ultrasound is a non-invasive test that can improve the accuracy of clinical evaluation. We will study whether use of heart, lung, and femoral and popliteal vein ultrasound performed at the patient bedside in addition to clinical evaluation can lead to a reduced length of stay in hospital, as well as shorten the time and number of tests performed, prior to the definitive diagnosis being made. The study will be

randomised (like flipping a coin) to compare "standard care" which does not routinely include ultrasound examination with the use of heart, lung and lower extremities veins ultrasound performed within 24 hours of admission to hospital.

3.2 INTRODUCTION

Internal medicine is well-known for its complexity. Most of the patients are elderly, with many comorbidities and requiring multiple medications. Typically, internal medicine physicians rely on the medical history and physical examination, collectively known as "clinical evaluation", to derive a differential diagnosis and initial management plan.

However, it is known that clinical evaluation alone is frequently inaccurate in determining the correct diagnosis. Incorporating a basic ultrasound examination performed at the patient's bedside, in addition to clinical evaluation increases diagnostic accuracy.(1, 2, 3, 4) This technique is termed "focused ultrasound" or "point-of-care ultrasound" and has become increasingly popular among other specialties such as anaesthesia and critical care. The admission diagnosis where addition of focused ultrasound examination may be most beneficial, is the "cardiopulmonary diagnosis" as it is frequently difficult to separate the cause of symptoms such as shortness of breath or collapse to either a cardiac or respiratory problem. Further. deep vein thrombosis may cause pulmonary embolus, but clinical evaluation for deep vein thrombosis is inaccurate. Although it is likely that focused ultrasound examination will improve diagnostic accuracy, which in turn will alter management decisions, there are no data identifying whether that will result in improved clinical outcomes such as length of stay.

3.3 BACKGROUND INFORMATION

Most of the literature has been performed in the settings of anaesthesia, critical care and emergency medicine. The outcomes are predominantly related to diagnostic and management changes. Heiberg et al.(5) have published a systematic review of use of focused cardiac ultrasound (FCU) in non-cardiac surgery anaesthesia and intensive care and reported a 41 - 51% change in diagnosis or management after FCU was performed. In anaesthesia, when it was performed as part of the pre-operative evaluation for a non-cardiac surgery, change in management was seen in 12% of asymptomatic patients if it was used as a screening tool, and between 54% and 82% when it was performed in participants suspected of having heart disease. In the emergency department, the use of focused ultrasound assessing heart, lungs, abdomen and/or lower limb veins led to a change in the primary diagnosis in 32.2% of patients and management plan in 50%.(6) In some life threating scenarios the impact is even higher. A change in therapy has been described in 89% of patients during a cardiopulmonary resuscitation(7), and an increase in accuracy for the correct diagnosis from 50% to 80% in patients with non-traumatic undifferentiated hypotension.(8)

Most of the literature investigates the use of focused cardiac ultrasound. However, the use of lung ultrasound has gained popularity, and is increasingly recognised as an useful diagnostic tool for the diagnosis of pneumonia, pneumothorax, pleural effusion and cardiogenic pulmonary oedema.(9-11) It is a helpful tool for rapidly identifying the aetiology of acute respiratory failure, and has a role in prognostic assessment in the intensive care setting, as underlying lung pathology is associated with ventilation time and mortality.(12) A 2-point compression ultrasound of the lower extremity vein has an excellent accuracy in the

detection of proximal deep venous thrombosis(13) and is becoming increasingly utilised as a bedside test in emergency medicine. This method involves merely femoral and popliteal vein examination, as the presence of isolated thrombi in proximal veins other than these two is very unlikely.(14)

Surprisingly, there are few data available in the internal medicine (general medicine) setting. Croft et al.(15) published a pilot study in internal medicine outpatients, in which adding a focused cardiac ultrasound to the standard evaluation altered diagnostic testing or medical therapy in 40% of the cases. Although there is good evidence for improved diagnostic accuracy of focused ultrasound in addition to clinical evaluation, and that leads to management changes, there are very few studies examining whether clinical outcomes such as mortality, morbidity or length of stay are improved by the use of focused ultrasound examination. From our research group, Canty DJ. et al. (16) published a pilot randomised trial in patients with hip fracture, in which focused cardiac ultrasound performed as part of the preoperative evaluation appeared to reduce the incidence of composite outcome of death, cardiac and renal complications by 39%. However, the study was a pilot study used to inform effect size and feasibility for a definitive pragmatic RCT, rather than provide a definitive answer.

It is plausible that improving diagnosis and altering management plans, may lead to improvement in the work flow and reduce of the length of hospital stay. There is limited data available to answer this question, but in emergency medicine a significant reduction in the emergency department length of stay was reported in two studies after the implementation of training programmes in point-of-care ultrasound.(17, 18) A further example is in patients with clinically important pericardial effusion, where a focused cardiac ultrasound in the emergency department reduced the time from presentation to pericardiocentesis from 70.2 to 11.3 hours. (19)

The patients who are admitted to general medical wards are often elderly with multiple co-morbidities. There are very few data investigating the use of focused ultrasound in this population. However, it is plausible that that these patients will not be that different from the emergency department, anaesthesia or intensive care populations, such that adding focused ultrasound to clinical evaluation is likely to alter diagnosis and management. Furthermore, it is plausible that if the correct diagnosis is made earlier in the admission time course, then length of stay could also be reduced. Improvements in workflow could also lead to reduced cost. In a general medical population with a “cardiopulmonary” admission diagnosis, the focused ultrasound may help to define whether the presenting problem is cardiac or respiratory in origin. By adding femoral and popliteal vein ultrasound, we can identify presence of proximal deep venous thrombosis, which could cause pulmonary embolus and explain patient symptoms, but also the finding of a vein thrombus by itself is clinically relevant as denotes change in the patient therapy and in some cases further studies to determine the aetiology. When routine ultrasound screening has been performed, 4.5 % of patients have an asymptomatic proximal deep venous thrombosis at the time of admission to the internal medicine ward.(20)

We aim to investigate whether use of focused cardiac, lung, and femoral and popliteal vein ultrasound in addition to clinical evaluation can reduce hospital length of stay. We will further aim to identify mechanisms such as time to definitive diagnosis, work flow and health care costs arising from investigations and bed stay.

4. Study Objectives

4.1 Hypothesis

The addition of focused cardiac, lung, and femoral and popliteal vein ultrasound to clinical evaluation will reduce the length of stay by at least 24 hours in a cohort of internal medicine patients with an admission diagnosis of a cardiopulmonary disease and expected to stay in hospital > 24 hours.

4.2 Study Aims

To identify whether focused cardiac, lung and femoral & popliteal ultrasound to clinical evaluation:

a. Reduced length of stay

b. Shortens the time to definitive diagnosis

c. Reduced the number of imaging or pathology tests performed prior to the definitive diagnosis is made

d. Identifies >10% of new significant heart or lung pathology

e. Is cost effective

4.3 Outcome Measures

A. Primary outcome: length of stay measured in hours form the time of admission to the medical unit until discharge from the hospital

B. Secondary outcomes

a. Time from admission to establishment of the definitive diagnosis (hours)

b. The frequency and type of new cardiopulmonary diagnoses that were not diagnosed prior to performance of the FCU and Lung ultrasound

c. The number and type of imaging and pathology investigations performed prior to establishment of the definitive diagnosis.

d. Cost will be based on bed stay and investigation costs for the Royal Melbourne Hospital

e. The proportion of participants where imaging cannot be obtained.

5. Study Design

5.1 Study Type & Design & Schedule

1. Trial design

Randomised controlled trial.

2. Cohort:

a. Adult participants ≥18 years will be included if they can provide informed consent and are admitted to an internal medicine unit at the Royal Melbourne Hospital with a cardiopulmonary diagnosis and are expected to remain in hospital >24 hours.

b. Exclusion: Participants will be excluded if they have already been admitted > 24 hours, are admitted for social rather than medical reasons, are unable to provide consent, are on contact or respiratory isolation, or have received a transthoracic echocardiogram within 4 weeks prior to admission, or a CT chest scan during the admission process prior to enrolment.

3. Location: Single-centre at the Royal Melbourne Hospital

4. The primary outcome will identify whether the ultrasound examinations leads to a meaningful reduction in length of stay. The secondary outcomes are designed to identify mechanisms to explain an effect if observed, such as the incidence of new diagnoses which were missed by clinical evaluation and the number of tests required prior to the definitive diagnosis being established.

5. Measurements – all measurements are re-identifiable

   a. Demographic information including gender, age, height and weight, prior medical conditions, medications, admission diagnosis

   b. Diagnosis information including the initial differential diagnosis, alternative diagnosis and the definitive diagnosis.

   c. Times including hospital length of stay, time to definitive diagnosis

   d. Investigations performed (type and number of tests)

      i. Imaging such as X-ray, CT, MRI, nuclear medicine and ultrasound including echocardiography

      ii. Pathology tests such as blood tests, urine tests, bacterial cultures or viral swabs.

   e. Cardiac, lung and vein pathology identified on the focused cardiac, lung, and femoral and popliteal vein ultrasound (intervention group only)

6. Data collection and handling

   a. Data will be collected through interview and extracted from hospital records.

   b. The treating medical staff (at least registrar level) will complete a custom diagnostic and management form on a daily basis (see CRF). Change in the custom form fields will be used to identify change.

   c. The number and type of tests will be identified from hospital records including online pathology and radiology services.

d. The definitive diagnosis will be reported by the treated medical team. The diagnosis will be verified against the discharge diagnosis.

e. All FCU lung, and femoral vein ultrasound images will be recorded in DICOM format. The images and report forms will be checked by an independent expert, as a quality assurance of the accuracy of the bedside ultrasound. Furthermore, if the participant undergoes comprehensive echocardiography or lung ultrasound or CT scan, the finding of the bedside examination will be cross checked against the formal investigations.

7. Time frames: participants will be enrolled within 24 hours of hospital admission and follow up concludes at hospital discharge.

8. No home visits

   This protocol will be used as part of a Master of Medicine degree for Dr Ximena Cid Serra at the University of Melbourne.

9. Provide a flowchart or table specifying visits, interventions and other relevant details

STUDY TABLE

	Assessment/Procedure	Within 24 hr of admission (Day 1)	Day 2	Day 3 and	Discharge day
Example procedures	Informed Consent	x			
	Demographic Information	x			
	Admission diagnosis and management plan	x			
	FCU, Lung and femoral and popliteal vein scan is allocated	x			
	Diagnosis, management and investigations form	x			x

5.2 Standard Care and Additional to Standard Care Procedures

Standard Care Procedures			Additional To Standard Care		
Procedure	Time/Visit	Dosage/Volume	Procedure	Time/Visit	Dosage/Volume
Clinical Evaluation by medical team	Daily until discharge	NA	Focused Cardiac, Lung and femoral and popliteal vein Ultrasound	Within 24 hours of admission to hospital	NA
Investigations	As determined by treating medical team	NA			

5.3 Randomisation

Randomisation is performed using internet software (https://www.sealedenvelope.com/simple-randomiser/v1/lists) in unequal block sizes of 4, 6 and 8. Allocation will be concealed in double sealed opaque envelopes. Allocation is revealed after informed consent is obtained by the researcher. The researcher is not blinded to allocation as the intervention (FCU, Lung, and femoral and popliteal ultrasound) is only performed in the intervention arm and cannot be blinded. Outcome data including length of stay or diagnostic and management decisions are determined by the treating medical staff and not under the influence of the investigators.

5.4 Study Methodology

This study will allocate participants to receive focused cardiac, lung, and femoral and popliteal ultrasound within 24 hours of admission (or not). All other aspects of care, including investigations performed, treatments provided and discharge planning is determined by the treating medical team. The intervention will provide additional information to the treating team only, with no direction about management, in keeping with a pragmatic trial design.

The intervention is focused cardiac, lung, and femoral and popliteal ultrasound, performed in accordance with the University of Melbourne Ultrasound Education Group training methods [ref]. The investigator will perform the ultrasound examinations and report these on the

iHeartScan and iLungScan report forms (University of Melbourne). **No index entries found.**See Appendix. The investigators are trained and competent to perform the examinations. All ultrasound examinations will be stored in DICOM format and checked by an expert investigator.

A diagnostic and management form (See CRF) will be completed before and after the ultrasound examination by the treating medical team. All other data is extracted from the hospital records and electronic databases.

6. Study Population

6.1 Recruitment Procedure

Potential participants will be identified from hospital admissions data within the preceding 24 hours. Specifically, the participants will have an initial admission diagnosis of "cardiopulmonary", and be admitted to one of the general medicine units (AMU, Gen Med 1-3)

Inclusion Criteria

Adult participants ≥18 years will be included if they can provide informed consent and are admitted to an internal medicine unit at the Royal Melbourne Hospital with a cardiopulmonary diagnosis and are expected to remain in hospital >24 hours.

6.2 Exclusion Criteria

Participants will be excluded if they have already been admitted for > 24 hours, are admitted for social rather than medical reasons, are unable to provide consent, are on contact or respiratory isolation, or have received a transthoracic echocardiogram within 4 weeks prior to admission, or a CT chest scan during the admission process prior to enrolment.

6.3 Consent

Individual written informed consent will be obtained.

7. Participant Safety and Withdrawal

7.1 Risk Management and Safety

The focused cardiac, lung and femoral vein ultrasound is a non-invasive procedure. The ultrasound is placed on the participants chest or leg with firm pressure and the scans take approxemetely 15 minutes to perform. The participant will be asked to lie on their left side and to sit up during the examination. The ultrasound itself does not pose any danger to the patient.

7.2 Handling of Withdrawals

Participants will be informed that they are free to withdraw from the study at any time. They will also be informed that their withdrawal will not affect the relationship between them and the treating doctors or the hospitals. They will be informed that any data collected to point of withdrawal will be used to prevent bias in the results, unless they specifically refuse that request. From the time of withdrawal, no further data will be acquired for that participant.

7.3 Replacements

We will not replace withdrawn participants. Rather, we have increased the sample size for the study to account for withdrawals.

8. Statistical Methods

8.1 Sample Size Estimation & Justification

The sample size estimates and statistical methods has been written by our consulting statistician, Dr Sandy Clarke, from the University of Melbourne Statistical Centre.

8.2 Power Calculations

The outcome of interest, hospital length of stay (LOS), is expected to be skewed and hence will be log transformed for the purposes of analysis. Control data was available from 2017, with a mean log based 10 LOS of 2.01 (equivalent to 103 hours on the original scale) and a standard deviation of 0.36.

In order to detect a clinically important reduction in LOS of ≥ 24 hours for the ultrasound group, based on the expected control value above and a common standard deviation on the log scale, power of 0.8 and size 0.05, 122 patients are required in each of the two treatment groups, which will be rounded up to a total sample size of 250 participants.

8.3 Statistical Methods To Be Undertaken

No censoring will be used for length of hospital stay. The primary outcome will be analyzed using Student's t-test on the log transformed LOS data in hours. Secondary outcomes will be analysed using parametric or non-parametric tests according to the type of data, whether the data is skewed, and whether repeated measures are used. For secondary endpoints, significance will be defined as $p<0.01$ to reduce the risk of Type 1 error.

9. Storage of Blood and Tissue Samples

9.1 Details of where samples will be stored, and the type of consent for future use of samples

N/A

10. Data Security & Handling

10.1 Details of where records will be kept & How long will they be stored

Paper records will be stored in a locked filing cabinet, in a locked room in the Department of Surgery, University of Melbourne. Electronic data are stored on password protected databases, available only to researchers involved in the study.

All paper records used during the study are kept after the project has been completed for a minimum of 5 years, and will be disposed of via shredding of all paper records, and deletion from databanks.

10.2 Confidentiality and Security

Only HREC authorised researchers who are involved in data collection and entry into the database will have access to the information. There are no separate student supervisors who are not associate investigators. Access to the information is provided by the Chief Investigator using login and password process.

Information will remain at the University of Melbourne. If the chief/principal investigator ceases to be engaged at the current organisation the principal investigator will inform the HREC that they will no longer be part of the study. Responsibility of the project will be handed over to an associate researcher working on the study already. HREC will be informed of relevant changes made.

No identifiable information will be used in any publication or presentation, and only group data will be published.

10.3 Ancillary data

Ultrasound images are stored on a password protected server and only accessible to the researchers.

11. APPENDIX

List of Attachments included:

Document Name	Version Number	Date (e.g., 18 January 2012)
CRF	1	20 May 2018
iHeart-iLung-veins form	1	28 May 2018

12. REFERENCES

1. Johnson BK, Tierney DM, Rosborough TK, Harris KM, Newell MC. Internal Medicine Point-of-Care Ultrasound Assessment of Left Ventricular Function Correlates with Formal Echocardiograpy. Journal of Clinical Ultrasound. 2016;44:92-9.
2. Razi R, Estrada JR, Doll J, Spencer KT. Bedside hand-carried ultrasound by internal medicine residents versus traditional clinical assessment for the identification of systolic dysfunction in patients admitted with decompensated heart failure. J Am Soc Echocardiogr. 2011;24(12):1319-24.
3. Sekiguchi H. Tools of the Trade: Point-of-Care Ultrasonography as a Stethoscope. Semin Respir Crit Care Med. 2016;37(1):68-87.
4. Bhagra A, Tierney DM, Sekiguchi H, Soni NJ. Point-of-Care Ultrasonography for Primary Care Physicians and General Internists. Mayo Clin Proc. 2016;91(12):1811-27.
5. Heiberg J, El-Ansary D, Canty DJ, Royse AG, Royse CF. Focused echocardiography: a systemic review of diagnostic and clinical decision-making in anaesthesia and critical care. Anaesthesia. 2016;71:1091-100.
6. Sasmaz MI, Gungor F, Guven R, Akyol KC, Kozaci N, Kesapli M. Effect of Focused Bedside Ultrasonography in Hypotensive Patients on the Clinical Decision of Emergency Physicians. Emerg Med Int. 2017;2017:6248687.
7. Breitkreutz R, Price S, Steiger HV, Seeger FH, Ilper H, Ackermann H, et al. Focused echocardiographic evaluation in life support and peri-resuscitation of emergency patients: a prospective trial. Resuscitation. 2010;81(11):1527-33.
8. Jones AE, Tayal VS, Sullivan DM, Kline JA. Randomized, controlled trial of immediate versus delayed goal-directed ultrasound to identify the cause of nontraumatic hypotension in emergency department patients. Crit Care Med. 2004;32(8):1703-8.
9. Chavez MA, Shams N, Ellington LE, Naithani N, Gilman RH, Steinhoff MC, et al. Lung ultrasound for the diagnosis of pneumonia in adults: a systematic review and meta-analysis. Respir Res. 2014;15:50.

10. Grimberg A, Shigueoka DC, Atallah AN, Ajzen S, Iared W. Diagnostic accuracy of sonography for pleural effusion: systematic review. Sao Paulo Med J. 2010;128(2):90-5.
11. Al Deeb M, Barbic S, Featherstone R, Dankoff J, Barbic D. Point-of-care ultrasonography for the diagnosis of acute cardiogenic pulmonary edema in patients presenting with acute dyspnea: a systematic review and meta-analysis. Acad Emerg Med. 2014;21(8):843-52.
12. Tierney DM, Boland LL, Overgaard JD, Huelster JS, Jorgenson A, Normington JP, et al. Pulmonary ultrasound scoring system for intubated critically ill patients and its association with clinical metrics and mortality: A prospective cohort study. Journal of clinical ultrasound : JCU. 2018;46(1):14-22.
13. Crisp JG, Lovato LM, Jang TB. Compression ultrasonography of the lower extremity with portable vascular ultrasonography can accurately detect deep venous thrombosis in the emergency department. Ann Emerg Med. 2010;56(6):601-10.
14. Adhikari S, Zeger W, Thom C, Fields JM. Isolated Deep Venous Thrombosis: Implications for 2-Point Compression Ultrasonography of the Lower Extremity. Ann Emerg Med. 2015;66(3):262-6.
15. Croft LB, Duvall WL, Goldman ME. A pilot study of the clinical impact of hand-carried cardiac ultrasound in the medical clinic. Echocardiography. 2006;23(6):439-46.
16. Canty DJ, Heiberg J, Yang Y, Royse AG, Margale S, Nanjappa N, et al. Pilot multi-centre randomised trial of the impact of pre-operative focused cardiac ultrasound on mortality and morbidity in patients having surgery for femoral neck fractures (ECHONOF-2 pilot). Anaesthesia. 2018;73(4):428-37.
17. Choi YJ, Jung JY, Kwon H. Effectiveness of education in point-of-care ultrasound-assisted physical examinations in an emergency department: A before-and-after study. Medicine (Baltimore). 2017;96(25):e7269.
18. Lin MJ, Neuman M, Rempell R, Monuteaux M, Levy J. Point-of-Care Ultrasound is Associated With Decreased Length of Stay in Children Presenting to the Emergency Department With Soft Tissue Infection. The Journal of emergency medicine. 2018;54(1):96-101.
19. Alpert EA, Amit U, Guranda L, Mahagna R, Grossman SA, Bentancur A. Emergency department point-of-care ultrasonography improves time to pericardiocentesis for clinically significant effusions. Clinical and experimental emergency medicine. 2017;4(3):128-32.
20. Ciuti G, Grifoni E, Pavellini A, Righi D, Livi R, Perfetto F, et al. Incidence and characteristics of asymptomatic distal deep vein thrombosis unexpectedly found at admission in an Internal Medicine setting. Thromb Res. 2012;130(4):591-5.

Statistical analysis plan -IMFCU study

Statistical analysis will be performed using the software SPPS version 27 (Statistical Package for the Social Sciences).

Distribution of demographic and baseline characteristics between groups will be analysed using Fisher test for categorical data and Student's t-test for ordinary data.

The primary outcome, length of hospital stay in hours, will be analysed using Student's t-test on log-transformed data to normalize skewed data and following an intention-to-treat approach. A cut-off of 30 days will be applied to avoid the effect of excessively extended hospital stays. Significance was defined as $p<0.05$. For patients who died in the hospital, death will be treated as hospital discharge for the primary analysis as the unpublished mortality rate for patients admitted to internal medicine wards at the Royal Melbourne Hospital is low (2.7%). Patients with missing data will not be included in the primary statistical analysis.

Because the introduction of Focused Clinical Ultrasound (FCU) was new to the internal medicine unit, we hypothesised that there would be a lead-in period where the FCU findings may not be accepted by the treating team, whereas as the physicians became more familiar with FCU, the finding would be more trusted and more likely to be acted upon. A sensitivity analysis will be performed on outcomes to compare the first half versus the second half of the participants.

The primary outcome will be analysed in pre-defined subgroups that we suspect a stronger effect of FCU: older than 75 years old, history of chronic heart failure, history of chronic obstructive pulmonary disease and patients with multiple comorbities.

Secondary outcomes will be analysed using parametric or non-parametric tests according to the type of data. All estimates will be reported with 95% confidence intervals.

1) Health costs:
   If the health costs data is skewed, it will be log-transformed to normalise its distribution and analysed using Student's t-test.

2) 30-day readmission data will be presented as dichotomic data and analysed using Fisher exact test.
3) The impact of FCU on the diagnosis and management will be reported in the intervention group only describing the number and proportion of participants.
