# Supplementary material for: Effect of a Multiorgan Focused Clinical Ultrasonography on Length of Stay in Patients Admitted With a Cardiopulmonary Diagnosis: A Randomized Clinical Trial
Source: JAMA Netw Open. 2021 Dec 21;4(12):e2138228. doi: 10.1001/jamanetworkopen.2021.38228 (PMC8693211; doi:10.1001/jamanetworkopen.2021.38228)
Supplement: Supplement 2. — eFigure. iLungScanTM and iHeartScanTM Report Filled With an Example eTable 1. Subgroup Analysis of the Effect of POCUS in the Health Costs eTable 2. Accuracy of Cardiac Clinical Ultrasound in 18 Patients Compared to Standard Echocardiography Performed by Cardiologists [file jamanetwopen-e2138228-s002.pdf]

Supplemental Online Content

Cid-Serra X, Royse A, Canty D, et al. Effect of a multiorgan focused clinical ultrasonography on length of stay in patients admitted with a cardiopulmonary diagnosis: a randomized clinical trial. *JAMA Netw Open*. 2021;4(12):e2138228. doi:10.1001/jamanetworkopen.2021.38228

eFigure. iLungScan™ and iHeartScan™ Report Filled With an Example

eTable 1. Subgroup Analysis of the Effect of POCUS in the Health Costs

eTable 2. Accuracy of Cardiac Clinical Ultrasound in 18 Patients Compared to Standard Echocardiography Performed by Cardiologists

This supplemental material has been provided by the authors to give readers additional information about their work.

© 2021 Cid-Serra X et al. *JAMA Network Open.*

eFigure. iLungScan^{TM} and iHeartScan^{TM} Report Filled With an Example

Legend: Not all the variable described in the forms are assessed and reported.

A brief sentence assessing deep venous thrombosis was added into the lung ultrasound report.

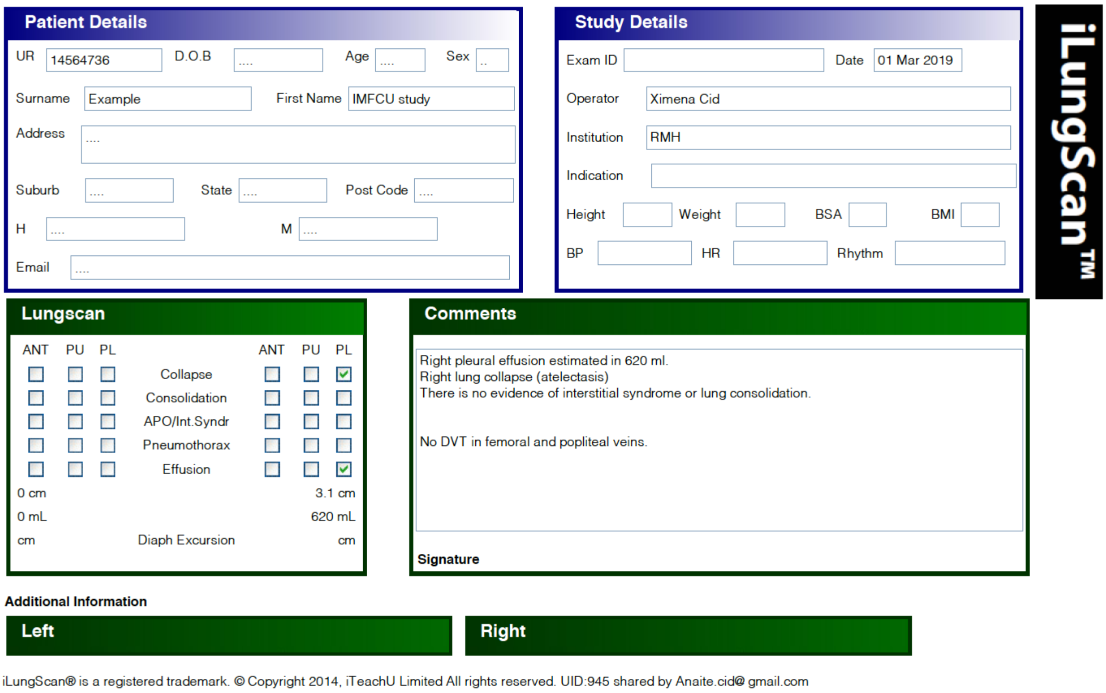

Patient Details

UR 14564736 D.O.B .... Age .... Sex ..

Surname Example First Name IMFCU study

Address ....

Suburb .... State .... Post Code ....

H .... M ....

Email ....

Study Details

Exam ID Date 01 Mar 2019

Operator Ximena Cid

Institution RMH

Indication

Height Weight BSA BMI

BP HR Rhythm

iLungScan™

Lungscan

ANT	PU	PL		ANT	PU	PL
☐	☐	☐	Collapse	☐	☐	☑
☐	☐	☐	Consolidation	☐	☐	☐
☐	☐	☐	APO/Int.Syndr	☐	☐	☐
☐	☐	☐	Pneumothorax	☐	☐	☐
☐	☐	☐	Effusion	☐	☐	☑
0 cm				3.1 cm		
0 mL				620 mL		
cm			Diaph Excursion	cm		

Comments

Right pleural effusion estimated in 620 ml.
Right lung collapse (atelectasis)
There is no evidence of interstitial syndrome or lung consolidation.

No DVT in femoral and popliteal veins.

Signature

Additional Information

Left **Right**

iLungScan® is a registered trademark. © Copyright 2014, iTeachU Limited All rights reserved. UID:945 shared by Anaite.cid@ gmail.com

Patient Details

UR: 14564608 | D.O.B: .... | Age: .... | Sex: ..

Surname: Example | First Name: IMFCU study

Address: ....

Suburb: .... | State: .... | Post Code: ....

H: .... | M: ....

Email: ....

Study Details

Exam ID: | Date (dd/mm/yyyy): 01 Mar 2019

Institution: RMH | Operator: Ximena Cid

○ TTE ○ TOE | Quality ◉ Good ○ Technically Difficult

Indication:

Height: | Weight: | BSA: | BMI:

BP: | HR: | Rhythm:

iHeartScan® Report

Haemodynamic echocardiography Assessment in Real Time
see www.heartweb.com.au

Ventricular Volume (M-mode / 2D)

○ Hypovolaemia ○ Normal ◉ Dilated

<3 | 3 - 5.6 | >5.6

<8 | 8 - 14 | >14

RV ◉ Normal ○ Increased

Systolic Function

○ Increased ○ Normal ◉ Decreased

> 44 | 28-44 | <28

> 65 | 50-65 | <50

RV ◉ Normal ○ Decreased

Ejection Fraction

LVEDD: 6.3 | LVESD:

LVEDA: | LVESA:

FS: | EF/FAC:

CO

LVOTd:

LVOT VTI:

HR:

CO: | CI:

Left Atrial Filling Pressure (Interatrial Septum Motion)

PSAX / A4Ch | ○ Low LA Pressure | ○ Normal LA Pressure | ◉ High LA Pressure

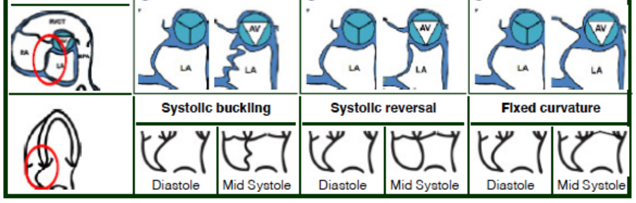

Valve Assessment

Examined	AV	MV	TV	PV
Not Significant	☑	☐	☐	☑
**Haemodynamically Significant**				
Stenosis	☐	☐	☐	☐
Regurgitation	☐	☑	☑	☐

☐ Pericardial Effusion

Haemodynamic State

	○ Normal	○ Empty	○ Vaso dilated	○ Primary Systolic Failure	○ Primary Diastolic Failure	◉ Systolic & Diastolic Failure	○ RV Failure
Volume	-	Decr	-	Incr	- / Decr	Incr	RV Incr
Systolic Function	-	- / Incr	Incr	Decr	-	Decr	RV Decr
Filling Pressure	-	Decr	-	-	Incr	Incr	Incr

Atria / PA pressure

LA diam: | RA diam:

LA area: 25 | RA area:

TR Vmax: | TVGr:

RAP: | RVSP:

HARTscan® is a limited transthoracic echocardiography study that is a qualitative rather than quantitative assessment.
It is intended to be completed in approximately 10 minutes. Additional more advanced courses are available.
This program is administered by The University of Melbourne, Australia. Visit www.heartweb.com.au

Comments

LV dilated and systolic function decreased
RV normal size and normal function
LA dilated with high filling pressure (fixed Inter atrial septum) suggesting LV diastolic dysfunction
Valves: significant MR and TR
No pericardial effusion
IVC 2 cm collapsing less than 50%

☐ Refer for full echocardiography study

Signature

HARTscan - Extended

AV

LVOTd | LVOT VTI | AV VTI

AVA | AVGp | AVGm

Dim Index | AI jet % | AI P1/2t

Ao/PA

Ao Root

Asc Ao

PA

MV

Radius | Scale | CW-MR | ERO

MV P1/2t | MVA | MVGp | MVGm

Diastolic Function

E | A | A dur | DT

S | D | S/D | pA dur

E' | E/A | E/E' | IVRT

LV

LVH

IVSWT | PWT

LV mass | LVi mass

HARTscan® is a registered trademark. © Copyright 2009. All rights reserved. This course and material is distrubuted by The University of Melbourne, Australia. UID:2454 shared by Anaite.cid@ gmail.com

eTable 1. Subgroup Analysis of the Effect of POCUS in the Health Costs.

Subgroup	Health costs by category	Mean difference in Australian dollars spent (SE)	Effect of POCUS in the costs	*P-value*
**Age <75 years**	Internal medicine unit	-229 (253)	Reduce 17.2%	0.58
	Internal medicine care	-327 (766)	Reduce 8.6%	0.97
	Pathology tests	-73 (140)	Reduce 13.9%	0.47
	Imaging tests	1.2 (84)	Increase 0.6%	0.4
	**Total in-hospital**	**-657 (1347)**	**Reduce 9.1%**	**0.95**
**Dementia**	Internal medicine unit	-294 (381)	Reduce 16.1%	0.65
	Internal medicine care	-1011(1056)	Reduce 18.0%	0.41
	Pathology tests	-176 (94)	Reduce 35.0%	0.14
	Imaging tests	+18 (81)	Increase 11.2%	0.06
	**Total in-hospital**	**-1847 (1826)**	**Reduce 18.5%**	**0.31**
**COPD**	Internal medicine unit	-421 (294)	Reduce 27.9%	0.15
	Internal medicine care	-360 (960)	Reduce 8.3%	0.4
	Pathology tests	-67 (96)	Reduce 17.7%	0.7
	Imaging tests	-32 (48)	Reduce 24.5%	0.28
	**Total in-hospital**	**-566 (1634)**	**Reduce 7.3%**	**0.73**
**Chronic cardiac failure**	Internal medicine unit	-167 (252)	Reduce 10.8%	0.62
	Internal medicine care	-15 (936)	Reduce 0.3%	0.98
	Pathology tests	-40 (110)	Reduce 8.7%	0.94
	Imaging tests	+53 (55)	Increase 34.9%	0.94
	**Total in-hospital**	**+91 (1952)**	**Increase 1.1%**	**0.73**

Abbreviation COPD: Chronic obstructive pulmonary disease. SE: standard error. POCUS: point-of-care ultrasound.

© 2021 Cid-Serra X et al. *JAMA Network Open.*

eTable 2. Accuracy of Cardiac Clinical Ultrasound in 18 Patients Compared to Standard Echocardiography Performed by Cardiologists

Variable	Sensitivity	Specificity
Left ventricle dilated	83%	83%
Left ventricle systolic dysfunction	100%	80%
Right ventricle dilated	100%	79%
Right ventricle dysfunction	75%	79%
Aortic stenosis	100%	100%
Aortic regurgitation	100%	94%
Mitral regurgitation	75%	100%
Tricuspid regurgitation	100%	87%
Pericardial effusion	100%	100%

The protocol used in this study reports only "significant" abnormalities, which correlates with moderate to severe alterations in standard echocardiography. Some variables' sensitivity or specificity might be affected by a discrepancy in the lesion's severity rather than a difference in a normal vs abnormal assessment

© 2021 Cid-Serra X et al. *JAMA Network Open.*
