# Supplementary material for: Effect of a Multiorgan Focused Clinical Ultrasonography on Length of Stay in Patients Admitted With a Cardiopulmonary Diagnosis: A Randomized Clinical Trial
Source: JAMA Netw Open. 2021 Dec 21;4(12):e2138228. doi: 10.1001/jamanetworkopen.2021.38228 (PMC8693211; doi:10.1001/jamanetworkopen.2021.38228)
Supplement: Supplement 3. — Data Sharing Statement [file jamanetwopen-e2138228-s003.pdf]

Data Sharing Statement

Cid-Serra. Effect of a Multiorgan Focused Clinical Ultrasonography on Length of Stay in Patients Admitted With a Cardiopulmonary Diagnosis. *JAMA Netw Open*. Published December 21, 2021. doi:10.1001/jamanetworkopen.2021.38228

Data
Data available: Yes
Data types: Deidentified participant data
How to access data: ximena.cid@hearweb.com
When available: With publication

Supporting Documents
Document types: None

Additional Information
Who can access the data: Researcher whose proposed use of the data has been approved
Types of analyses: systematic reviews and cost analysis
Mechanisms of data availability: After approval of a proposal and a signed data access agreement.
